# Preparation and Properties of Physical Gel on Medical Titanium Alloy Surface

**DOI:** 10.3390/gels9070558

**Published:** 2023-07-08

**Authors:** Yu Fu, Qingrong Wu, Wanying Yang, Jiaqi Wang, Zechen Liu, Hao Shi, Shouxin Liu

**Affiliations:** Key Laboratory of Applied Surface and Colloid Chemistry, Ministry of Education, School of Chemistry and Chemical Engineering, Shaanxi Normal University, Xi’an 710119, China; fy2247127430@163.com (Y.F.); wuqingrong123456@163.com (Q.W.); yangwy15935903470@163.com (W.Y.); wjqsxdtdx@163.com (J.W.).; qi363396@163.com (Z.L.); 15705119010@163.com (H.S.)

**Keywords:** hydrogels, medical titanium alloy, dopamine, mechanics

## Abstract

Medical titanium alloy Ti-6Al-4V (TC4) has been widely used in the medical field, especially in human tissue repair. However, TC4 has some shortcomings, which may cause problems with biocompatibility and mechanical compatibility in direct contact with the human body. To solve this problem, physical gels are formed on the surface of TC4, and the storage modulus of the formed physical gel matches that of the human soft tissue. 2-bromoisobutyryl bromide (BIBB) and dopamine (DA) were used to form initiators on the surface of hydroxylated medical titanium alloy. Different initiators were formed by changing the ratio of BIBB and DA, and the optimal one was selected for subsequent reactions. Under the action of the catalyst, L-lactide and D-lactide were ring-opened polymerized with hydroxyethyl methacrylate (HEMA), respectively, to form macromolecular monomers HEMA-PLLA_29_ and HEMA-PDLA_29_ with a polymerization degree of 29. The two macromolecular monomers were stereo-complexed by ultrasound to form HEMA-stereocomplex polylactic acid (HEMA-scPLA_29_). Based on two monomers, 2-(2-methoxyethoxy) ethyl methacrylate (MEO_2_MA) and oligo (ethylene oxide) methacrylate (OEGMA), and the physical crosslinking agent HEMA-scPLA_29,_ physical gels are formed on the surface of TC4 attached to the initiator via Atom Transfer Radical Addition Reaction (ATRP) technology. The hydrogels on the surface of titanium alloy were characterized and analyzed by a series of instruments. The results showed that the storage modulus of physical glue was within the range of the energy storage modulus of human soft tissue, which was conducive to improving the mechanical compatibility of titanium alloy and human soft tissue.

## 1. Introduction

Medical titanium alloy is a class of biomedical materials used in surgical implants and orthopedics. However, a series of problems such as inflammation [1], biological poisoning [2], and bacterial infection [3] will occur after the implantation of medical titanium alloy into the human body; therefore, in the process of developing and utilizing biomedical titanium alloy materials, people have always focused on improving the biocompatibility and mechanical compatibility of TC4. By studying the mechanism of action between titanium alloy and human body fluids and soft tissues, people are constantly looking for polymer materials [4,5,6], small molecular materials [7,8], and trace elements [9] to modify the surface of titanium alloy. Currently, the commonly used modification methods include mechanical surface modification [10], physical surface modification [11,12,13], and chemical surface modification. Chemical surface modification is the most common surface modification method, including the acid–base corrosion method [14], ATRP method [15,16,17], anodic oxidation method [18], sol-gel method [19], and so on. ATRP method is widely used in material surface modification due to its mild reaction conditions, simple operation, and wide monomer selectivity.

Hydrogels are similar to human soft tissues in terms of their mechanical and chemical properties. The formation of hydrogels on TC4 is conducive to improving the biocompatibility [20,21,22,23] and mechanical compatibility [24] of medical titanium alloys. Hydrogels can be divided into chemical and physical hydrogels according to the crosslinking mode. Compared with chemical hydrogels, physical hydrogels do not require chemical crosslinking agents and catalysts. Physical hydrogels are characterized by low toxicity and mild reaction conditions [25,26,27,28,29,30,31], but the mechanical strength of physical hydrogels needs to be improved. Polylactic acid (PLA) is a degradable material with strong mechanical strength and good biocompatibility. In recent years, PLA has been widely concerned [32,33]. PLA has pairs of enantiomers, including left-handed polylactic acid (PLLA) and right-handed polylactic acid (PDLA). PLLA and PDLA can be used as physical crosslinking agents in the complexation ratio of 1:1. Adding polylactic acid to physical hydrogels can improve the weakness of poor mechanical strength of physical gels. 2-(2-methoxyethoxy) ethyl methacrylate (MEO_2_MA) and oligo (ethylene oxide) methacrylate (OEGMA) are analogs of polyethylene glycol (PEG), and the polymer P(MEO_2_MA-*co*-OEGMA) has the properties of PEG. The mechanical properties of hydrogels can be changed by adding PEG [34].

In previous work [35], our research group modified TC4 with BIBB and DA and then formed hydrogel on its surface. After testing the binding ability of titanium alloy and hydrogel, it was found that hydrogel and titanium alloy had strong adhesion, indicating that the initiator had a strong anchoring force. In this paper, TC4 is modified by BIBB and DA. In order to make the initiators on the surface of titanium alloy more uniform, different initiators were formed by changing the ratio of BIBB and DA, and the optimal one was selected for the subsequent reaction. Under the action of the catalyst, L-lactide and D-lactide were ring-opened polymerized with HEMA, respectively, to form macromolecular monomers HEMA-PLLA_29_ and HEMA-PDLA_29_ with a polymerization degree of 29. The two macromolecular monomers were stereo-complexed by ultrasound to form HEMA-scPLA_29_. Based on the monomers MEO_2_MA, OEGMA, and the physical crosslinking agent HEMA-scPLA_29_, physical crosslinking hydrogels were formed on TC4 with an initiator by ATRP technology.

## 2. Results and Discussion

### 2.1. Synthesis

**Scheme 1:**
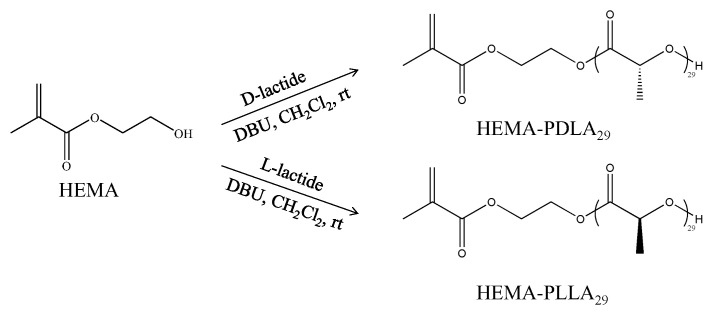
Synthesis of macromolecular monomer HEMA-PDLA_29_(PLLA_29_).

**Scheme 2:**
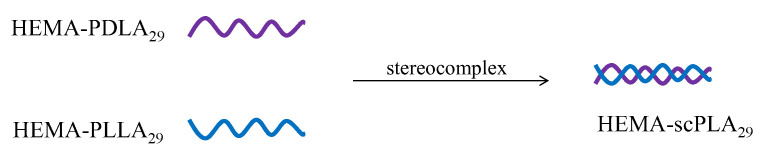
Schematic diagram of the synthesis of the physical crosslinking agent HEMA-scPLA_29_.

**Scheme 3:**
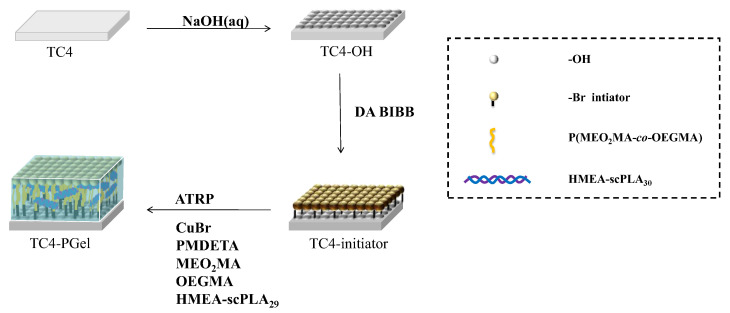
Schematic diagram of the synthesis of physical hydrogels PGel on the surface of medical titanium alloys.

Under the action of the catalyst, L-lactide and D-lactide were ring-opened polymerized with HEMA, respectively, to form macromolecular monomers HEMA-PLLA_29_ and HEMA-PDLA_29_ with a polymerization degree of 29, as shown in Scheme 1. The two macromolecular monomers were stereo-complexed by ultrasound to form HEMA-scPLA_29_, as shown in Scheme 2. Based on the monomers MEO_2_MA, OEGMA, and the physical crosslinking agent HEMA-scPLA_29_, physical crosslinking hydrogels were formed on the surface of TC4 with the initiator by ATRP technology, as shown in Scheme 3. Different physical crosslinkers have different physical crosslinking points and different pore sizes. The mechanical properties of hydrogels can be adjusted by changing the content of the physical crosslinking agent. Three different hydrogels were synthesized, and the content of the physical crosslinking agent HEMA-scPLLA_29_ was used as a variable. The properties of hydrogels are also studied. The specific feeding materials are shown in Table 1.

### 2.2. Structure Characterization of Macromolecular Monomers and Initiators

**Figure 1:**
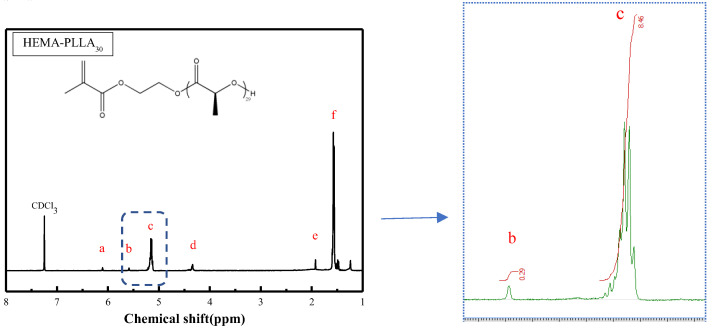
The ^1^H NMR spectrum of HEMA-PLLA_29_.

Figure 1 is the nuclear magnetic spectrum of macromolecular monomer HEMA-PLLA_29_. The specific proton peak shift value can be seen from the ^1^H NMR spectrum. The peaks of 6.10 ppm (a) and 5.59 ppm (b) are attributed to the proton peak of the carbon-carbon double bond; the peaks of 5.12–5.23 ppm (c) are from the methylene proton peak in –C=O)–CH(CH_3_)–; the 4.30–4.38 ppm (d) peaks come from the methylene proton peak in –(C=O)OCH_2_CH_2_O–; the 1.94 ppm (e) peak comes from the methyl proton peak in –(CH_3_)C=CH_2_; The 1.42–1.66 ppm (f) peaks come from the proton peak of the methyl group in –(C=O)–CH(CH_3_)–. HEMA-PDLA_29_ and HEMA-PLLA_29_ are identical to each other. ^1^H NMR spectra demonstrated the successful synthesis of HEMA-PDLA_29_ and HEMA-PLLA_29_.

The formula for calculating the number *n* of LA cells in HEMA-PLLA_29_(PDLA_29_):

(1) $$n=\frac{A_{c}}{A_{b}}$$

In the formula, $A_{c}$ and $A_{b}$ represent the integral area of c and b, respectively; n is the number of LA units of HEMA-PLLA_29_ (PDLA_29_); $A_{c}$ = 8.46, $A_{b}$ = 0.29, so *n* = 29.2 ≈ 29 [36].

**Figure 2:**
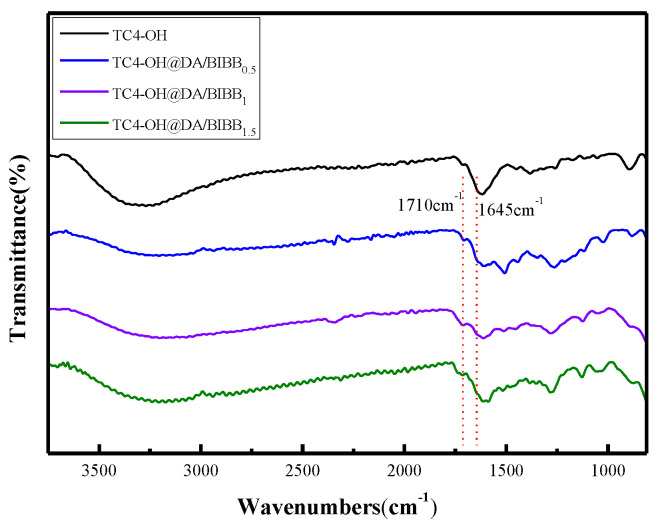
Infrared spectra of initiator TC4−OH@DA/BIBB_n_ (the molar ratio of DA/BIBB was 1:0.5, 1:1, and 1:1.5, respectively).

Figure 2 shows the Fourier transform infrared spectra of hydroxylated titanium alloy TC4-OH and a series of initiators TC4-initiator. FT-IR was used to characterize the surface of TC4-OH and a series of initiators TC4-initiator. In the FT-IR spectrum of TC4-OH, a wide and strong hydroxyl peak appeared at 2900–3670 cm^−1^, indicating that the hydroxyl group was successfully grafted onto TC4. In the FT-IR spectra of initiators TC4-OH@DA/BIBB_0.5_, TC4-OH@DA/BIBB_1_, TC4-OH@DA/BIBB_1.5_, the characteristic absorption peaks appeared at 1710 cm^−1^, 1645 cm^−1^, 1600 cm^−1^, 1510 cm^−1^, and 1250 cm^−1^. The ester formed by the reaction of the hydroxyl group in DA with BIBB showed a weak absorption peak at 1710 cm^−1^. The amide formed by the reaction of DA and BIBB showed an absorption peak at 1645 cm^−1^. The characteristic absorption peaks of the aromatic ring are 1600 cm^−1^, 1510 cm^−1^, and 1250 cm^−1^. The appearance of these peaks proved that different proportions of DA and BIBB could successfully synthesize modified polydopamine initiator on TC4 [37].

### 2.3. Micro and Macro Analysis of Initiator Surface Morphology

**Figure 3:**
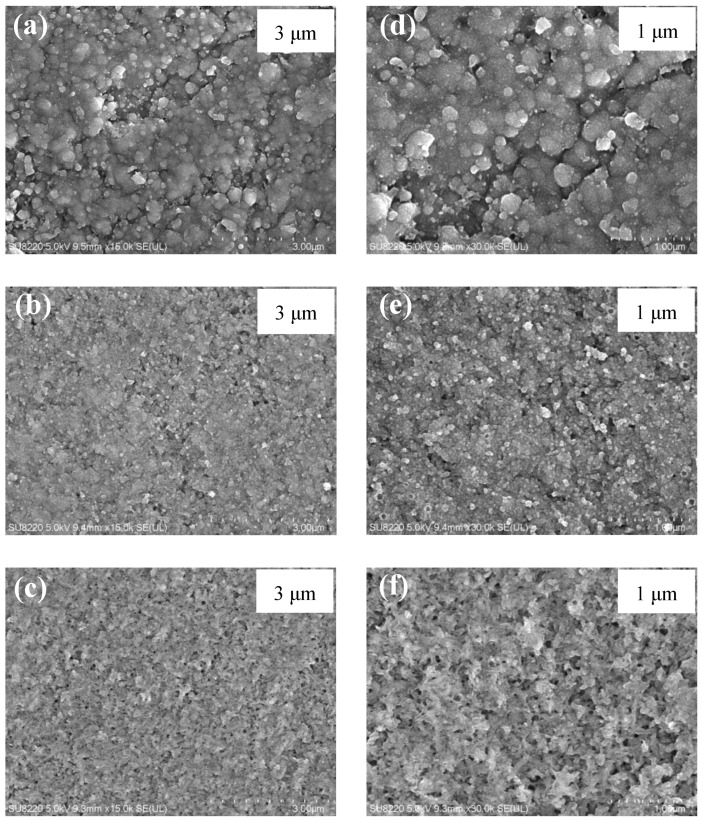
Microstructure of initiators TC4-OH@DA/BIBB_0.5_ (**a**), TC4-OH@DA/BIBB_1_ (**b**), and TC4-OH@DA/BIBB_1.5_ (**c**) at the same multiples. TC4-OH@DA/BIBB_0.5_ (**d**), TC4-OH@DA/BIBB_1_ (**e**), and TC4-OH@DA/BIBB_1.5_ (**f**) were the microstructure of initiators at the same multiples [enlarge the local parts of (**a**–**c**)].

Figure 3 shows high-resolution field emission SEM images of the initiator TC4-OH@DA/BIBB_n_ (molar ratios of DA/BIBB were 1:0.5, 1:1, and 1:1.5, respectively). Figure 3a–c show the microstructure of initiators at the same multiple, and Figure 3d–f show the microstructure of initiators at the same multiple (enlarge the local parts of Figure 3a–c). As can be seen from the figure, there are cracks on the surface of TC4-OH@DA/BIBB_0.5_, which will affect the life of the surface-modified materials. With the increasing proportion of BIBB content, the crack decreased gradually. In addition, considering the introduction of more initiators, TC4-OH@DA/BIBB_1.5_ was selected for the following reaction.

### 2.4. X-ray Diffraction Analysis of Physical Gels

**Figure 4:**
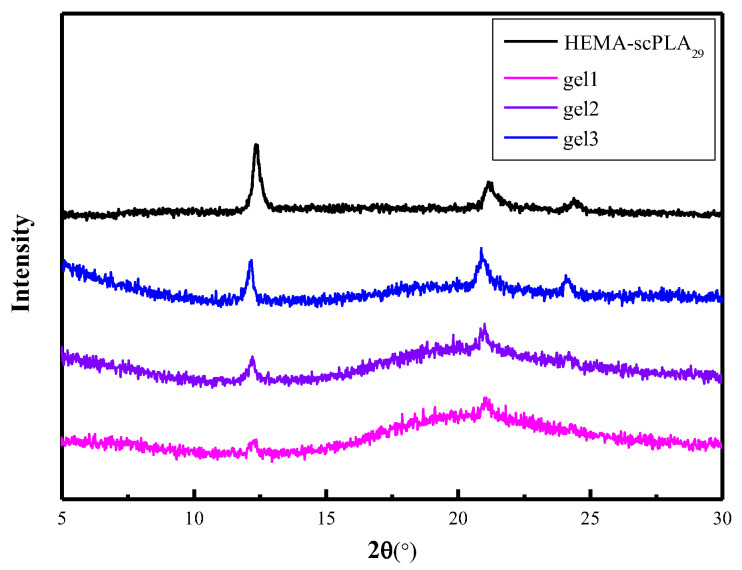
XRD patterns of stereocomplex macromolecular monomers and physical hydrogels PGel.

The stereocomplex structures in physical hydrogels were verified by an X-ray diffractometer (XRD). Figure 4 shows the XRD patterns of stereocomplex macromolecular monomers HEMA-scPLA_29_, PGel1, PGel2, and PGel3. In the diffraction pattern of macromolecular monomer, diffraction peaks appear at 2*θ* = 12°, 21°, and 24°, which is characteristic of PLA stereostructure [38]. The appearance of characteristic peaks indicates the successful synthesis of the stereocomplex. Compared with the XRD pattern of PGel1, PGel2, and PGel3, this series of gels also show diffraction peaks at the same position. With the gradual increase of physical crosslinking agents, the diffraction peak is increasingly obvious. This demonstrated the successful synthesis of hydrogels with different numbers of physical crosslinking points.

### 2.5. Morphological Analysis of Physical Gels

**Figure 5:**
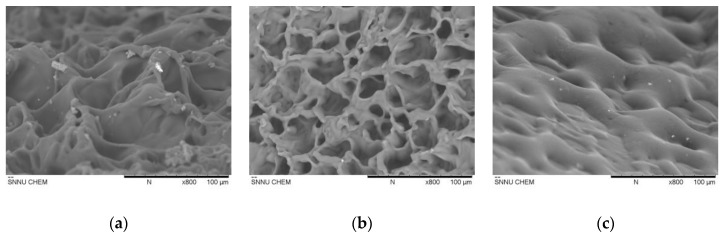
SEM images of physical hydrogels PGel1 (**a**), PGel2 (**b**), PGel3 (**c**).

Hydrogels have three-dimensional network structures, and the surface structure can be clearly observed under a low-power electron microscope. A TM3030 bench scanning electron microscope was used to observe the morphology of three kinds of hydrogels under the same magnification microscope. The size of a three-dimensional network of hydrogels could be judged by the size of the aperture. Figure 5 shows the SEM images of PGel1 (a), PGel2 (b), and PGel3 (c) at the same multiples. It can be seen from the figure that these three gels have a three-dimensional network structure. It can also be seen from the figure that the pores of PGel1, PGel2, and PGel3 gradually decrease. This is because the more the content of the physical crosslinking agent, the more interaction points in the hydrogel, the greater the crosslinking density, and the smaller the pore size. In contrast, PGel3, with the highest content of physical crosslinking agent, has the smallest pore diameter, and PGel1, with the lowest content of physical crosslinking agent, has the largest pore diameter.

### 2.6. Mechanical Properties of Physical Gels

Forming a gel on TC4 is equivalent to building a bridge between human soft tissue and objects with a large storage modulus, acting as a buffer. The storage modulus of the formed gel should be between the storage modulus of the human soft tissue (1–100 kPa) [39].

**Figure 6:**
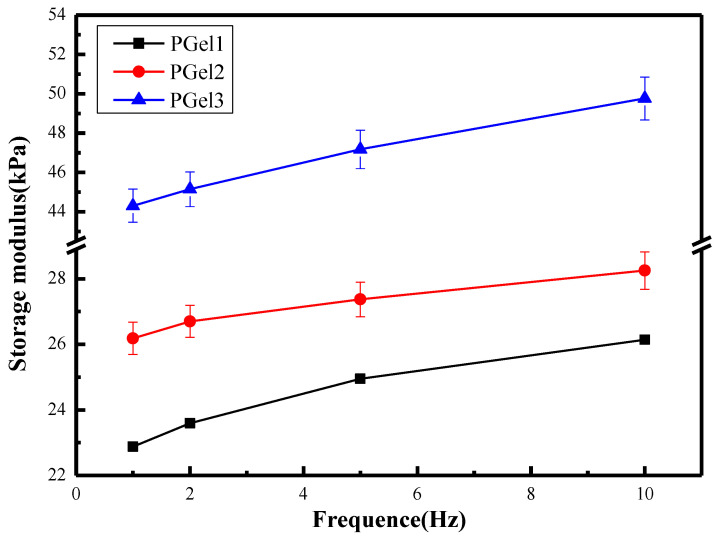
The variation curves of physical hydrogels PGel storage modulus with frequency.

The dynamic mechanic’s analyzer can be used to measure the energy storage modulus and loss tangents. Figure 6 shows the variation curve of the storage modulus of PGel1, PGel2, and PGel3 with frequency. When the frequency is 10 Hz, the storage modulus of PGel1, PGel2, and PGel3 are 26.14 kPa, 28.25 kPa, and 49.76 kPa, respectively. The storage modulus of three gels is matched with that of human soft tissue. The results showed that the hydrogels based on the monomers MEO_2_MA and OEGMA, and the physical crosslinking agent HEMA-scPLA_29_ could be used to improve the surface mechanical compatibility of TC4. In addition, it can also be seen from the figure that the storage modulus of these three gels increases with the increase of physical crosslinker content. This is because when other conditions remain unchanged, the content of the physical crosslinking agent in the gel will make the three-dimensional network structure of the gel different. PGel3 has the highest content of physical crosslinking agent, and the crosslinking density is the largest. Under the same conditions, PGel3 has the tightest three-dimensional network structure and is not easily broken; therefore, it has the largest storage modulus and the best mechanical properties.

**Figure 7:**
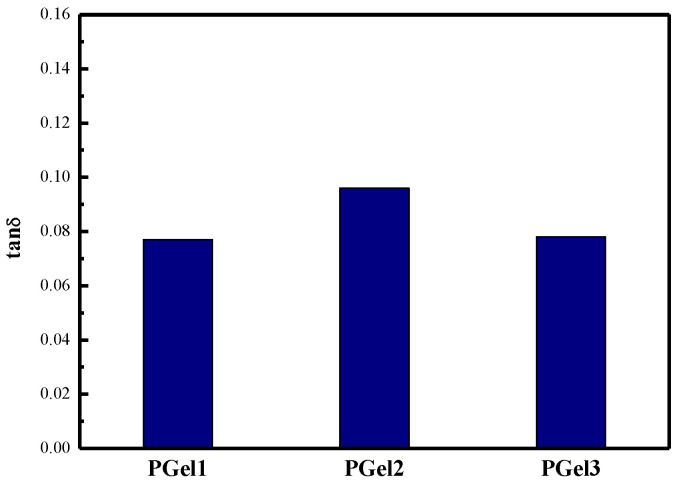
The loss tangents of physical hydrogels PGel at a frequency of 10 Hz.

Figure 7 shows the loss tangent (tanδ) of PGel1, PGel2, and PGel3 at a frequency of 10 Hz. The loss tangent is a parameter that indicates the viscoelasticity of a material. The loss tangent value is large, and the material is mainly viscosity; its value is small and the material is mainly elastic. The tanδ of the three gels were 0.077, 0.096, and 0.078, respectively. The tanδ are all very small, indicating that the hydrogels on TC4 are mainly elastic under external forces.

## 3. Conclusions

In this paper, different proportions of dibromoisobutyl bromide and dopamine were used to form SI-ATRP initiators on the surface of medical titanium alloy. Comparing different proportions of initiators, the best one for the subsequent reaction was chosen. Under the action of the catalyst, L-lactide and D-lactide were ring-opened polymerized with HEMA, respectively, to form macromolecular monomers HEMA-PLLA_29_ and HEMA-PDLA_29_ with a polymerization degree of 29. The two macromolecular monomers were stereo-complexed by ultrasound to form HEMA-scPLA_29_. Based on two monomers, MEO_2_MA and OEGMA, and the physical crosslinking agent HEMA-scPLA_29,_ physical gels are formed on the surface of TC4 attached to the initiator via ATRP technology. The results show that the storage modulus of the hydrogel formed by titanium alloy lies in the range of human soft tissue, which is conducive to improving the mechanical compatibility of medical titanium alloy. Among the three gels, PGel3, with the highest degree of crosslinking, has the largest energy storage modulus and the strongest ability to maintain its shape; therefore, PGel3 is the best choice. In addition, hydrogels are substances with a three-dimensional network structure and loose pores. There are also related applications in drug delivery and sustained release. Therefore, a hydrogel layer is introduced on the surface of medical titanium alloys, which has the potential to accelerate cell proliferation by loading antibacterial agents and growth factors in the gel.

## 4. Materials and Methods

### 4.1. Materials

Titanium alloy (TC4) was purchased from Baotai Group Co., Ltd., Shaanxi, China. 2-(2-methoxyethoxy) ethyl methacrylate (MEO_2_MA, 95%, Mn = 188.22 g·mol^−1^), oligoethylene glycol methyl ether methacrylate (OEGMA, 95%, Mn = 475 g·mol^−1^), and L-lactide (99%) were purchased from Shanghai Maclin Biochemical Technology Co., Ltd., Shanghai, China. D-lactide (99%) was purchased from China Aladdin Chemical Reagent Co., Ltd., Shanghai, China; 1,8-diazadicyclodecundecane-7-ene (DBU, 98.0%), hydroxyethyl methacrylate (HEMA, 99.0%), and 2-bromoisobutyl bromide (BIBB, 98.0%) were purchased from China Beijing Bailingwei Technology Co., Ltd., Beijing, China. Dopamine (DA, 95.0%) was purchased from Jiangsu Aikang Biomedicine R&D Co., Ltd., Wuhan, China. N,N′-dimethylformamide (DMF, 99.8%) was purchased from Shanghai Anhui Zeseng Co., Ltd., Shanghai, China. Dichloromethane and tris (hydroxymethyl) aminomethane (tris) were purchased from Guangdong Guanghua Technology Co., Ltd., Shantou, China. Triethylamine (TEA, 99.0%) was purchased from Shanghai Sinophosphoric Chemical Reagent Co., Ltd., China. N,N,N′,N′,N″-pentamethyldiethylenetriamine (PMDETA, 98%) was purchased from Shanghai Jingpure Reagent Co., Ltd., Shanghai, China.

### 4.2. Methods

#### 4.2.1. Preparation of TC4-OH

The polished TC4 was ultrasonically cleaned with acetone, ethanol, and distilled water successively for 10 min. Then, TC4 was placed in a 5 mol/L NaOH solution at a reaction temperature of 60 °C for 24 h. The surface of Ti was cleaned with distilled water and ethanol, and then the Ti sheets were dried under pressure at 45 °C for 24 h to obtain TC4-OH.

#### 4.2.2. Preparation of TC4-Initiator

In the nitrogen atmosphere, dopamine (200.0 mg, 1.05 mmol) was dissolved in N,N′-dimethylformamide (DMF, 10 mL). 2-bromoisobutyryl bromide (BIBB, 0.065 mL, 0.525 mmol) was added drop by drop under an ice water bath, followed by triethylamine (0.075 mL, 0.525 mmol). After the ice bath was removed, the mixture was stirred at room temperature in a nitrogen atmosphere for another 3 h. The mixture was then transferred to a beaker with a tris buffer solution (480 mg tris and 100 mL deionized water). A few more pieces of TC4-OH were added to this new mixture and stirred for 24 h. It was then washed with deionized water and dried under pressure at 60 °C for 24 h. The initiator was synthesized according to DA:BIBB = 1:0.5. The DA amount was changed, and the other reactants’ amount was kept. DA (200.0 mg, 1.05 mmol) was replaced by DA (100.0 mg, 0.525 mmol), and another proportional initiator (DA:BIBB = 1:1) was synthesized according to the above steps. The amount of DA was changed to DA (66.7 mg, 0.35 mmol), and another proportional initiator (DA:BIBB = 1:1.5) was synthesized according to the above steps. Three TC4-initiators were obtained.

#### 4.2.3. Synthesis of Macromolecule HEMA-PLLA_29_(PDLA_29_)

The whole process was conducted in a nitrogen atmosphere. HEMA (56 μL, 0.5 mmol) and L-lactide (1 g, 6.9 mmol) were placed in a clean and dry 50 mL three-necked flask. A total of 25 mL dichloromethane (DCM) was added. All solids in the flask were dissolved by stirring with magnetons, after which the catalyst DBU (100 μL, 0.65 mmol) was added. The reaction was carried out at room temperature for 12 h, and then benzoic acid was added for quenching. The reaction mixture was then precipitated with n-ethane in an ice water bath and washed twice. Finally, the mixture was dried for 12 h to remove the solvent and obtain the macromolecular monomer HEMA-PLLA_29_. The synthesis method of HEMA-PDLA_29_ is consistent with that of HEMA-PLLA_29_, which is not described here.

#### 4.2.4. Preparation of Hydrogels on TC4

The physical gels are prepared on TC4, and the synthesis of PGel1 is taken as an example. A total of 0.025 g HEMA-PLLA_29_ and 0.025 g HEMA-PLLA_29_ were placed into a 10 mL flask. Then, 2.5 mL of tetrahydrofuran was added to dissolve macromolecular monomers and sonicated for 1 h in an ultrasound machine to stereo-complexate. The solvent in the flask was dried by a rotary evaporator. The prepared physical crosslinking agent HEMA-scPLLA_29_ was placed in the fume hood for use.

The hydrogel was prepared by the ATRP technique. In 50 mL of dried Shrek tube, the monomers OEGMA (0.296 mL, 0.673 mmol) and MEO_2_MA (0.704 mL, 3.815 mmol), the prepared physical crosslinking agent HEMA-scPLLA_29_, 0.1 mL MeOH, and 0.1 mL H_2_O were added. The closed system was frozen and thawed three times under nitrogen. Then, 2 μL N,N,N′,N′,N″-pentamethyldiethylenetriamine (PMDETA) and a small amount of the catalyst CuBr were added under continuous nitrogen flow. After 5 min, the TC4-initiator (DA:BIBB = 1:1.5) was added. The reaction mixture was polymerized and cross-linked without oxygen in an oil bath at 65 °C. The synthesized sample was removed and soaked in deionized water for 12 h. The deionized water was replaced regularly to remove unreacted monomers and impurities. PGel1 was successfully produced on TC4. The method of synthesizing PGel2 and PGel3 was the same as above.

### 4.3. Structure Characterization

Nuclear magnetic resonance (^1^H NMR): the 400 MHz NMR spectrometer of the JNM-ECZ400S/L1 model made by Nippon Electronics Co., Ltd. was used to determine macromolecular monomers. Before the measurement, the sample to be tested was dissolved by CDCl_3_ and placed in a nuclear magnetic tube.

Attenuated Total Refraction Fourier Transform Infrared (ATR-FTIR): TC4 surface activated by hydroxyl and the TC4 surface modified with different proportions of BIBB and DA were measured by Frontier ATR-FTIR by PerkinElmer. The sample was kept dry before measurement.

### 4.4. X-ray Diffraction Analysis of Physical Gels

The D8 Advance powder X-ray diffractometer (XRD) manufactured by Bruker of Germany was analyzed by Ni-filtered Cu Kα (λ = 0.154 nm) at a room temperature of 25 °C. The instrument operates at a voltage of 40 kV and a current of 40 mA. The scanning rate of 2θ is 2°/min, and the scanning range is 5°~30°.

### 4.5. The Scanning Electron Microscope (SEM) Analysis of the Initiators and Physical Gels

The surface morphologies of TC4-initiator with different proportions were observed by SU8220 high-resolution field emission scanning electron microscope. In order to increase the conductivity of the sample, it is necessary to spray gold before the sample determination. However, the resolution of high-resolution field emission scanning electron microscope is higher, and the gold particles are more obvious. If titanium alloy is sprayed with too much gold, it will affect the observation of morphology; therefore, it is necessary to control the gold spraying time. It was sprayed 10 s before measurement.

The micromorphologies of three kinds of gels were observed by TM3030 benchtop scanning electron microscope under the same low-power microscope. Sample preparation method: three hydrogels were immersed in deionized water at room temperature to achieve expansion equilibrium. The three hydrogels were rapidly cooled with liquid nitrogen and then dried in a freeze-dryer. In order to improve the electrical conductivity of the sample, it was necessary to spray gold for 30 s before the sample measurement.

### 4.6. Mechanical Properties Analysis of the Physical Gels

Three hydrogels with different numbers of physical crosslinking points were synthesized at TC4. The energy storage modulus and loss tangent of a series of hydrogels were measured by a dynamic mechanics analyzer (DMA, 242E) in standard mode. In standard mode, the parameters are set as follows: the temperature is 37 °C, and the amplitude reaches 7% of the sample length. The duration of each loading is 15 min, and the cycle test is 5 times. Before testing, the sample should be soaked in deionized water at 37 °C for 12 h until swelling equilibrium.

## Figures and Tables

**Table 1:** Synthesis data of physical hydrogels PGel.

Sample	*m*(HEMA-PDLA_29_) (g)	*m*(HEMA-PLLA_29_) (g)	*n*(MEO_2_MA) (mmol)	*n*(OEGMA) (mmol)
PGel1	0.025	0.025	3.815	0.673
PGel2	0.1	0.1	3.815	0.673
PGel3	0.15	0.15	3.815	0.673

The ratio between *n*(MEO_2_MA) and *n*(OEGMA) is 85:15.

## Data Availability

No data availability statement.
